# Predictors of Sentinel Lymph Node Metastasis and External Validation of the MSKCC Nomogram in Breast Cancer: A Retrospective Single-Center Cohort Study

**DOI:** 10.3390/diagnostics16172823

**Published:** 2026-09-02

**Authors:** Darko Zdravković, Barbara Loboda, Simona Petricevic, Milan Gojgic, Natasa Colakovic, Dragana Bjelica, Igor Nađ, Vladimir Milosavljević, Svetlana Opric, Aleksandar Jovanović, Višnja Stojanović, Ljiljana Marković-Denić, Vladimir Nikolić

**Affiliations:** 1 Department of Surgical Oncology, University Hospital Medical Center “Bežanijska Kosa”, 11070 Belgrade, Serbialoboda.barbara@bkosa.edu.rs (B.L.); petricevic.simona@bkosa.edu.rs (S.P.); colakovic.natasa@bkosa.edu.rs (N.C.); jovanovic.aleksandar@bkosa.edu.rs (A.J.); stojanovic.visnja@bkosa.edu.rs (V.S.); 2 Medical Faculty, University of Belgrade, 11000 Belgrade, Serbia; 3 Department of Diagnostic Imaging Center, University Hospital Medical Center “Bežanijska Kosa”, 11070 Belgrade, Serbia; bjelica.dragana@bkosa.edu.rs; 4 Department of General and Abdominal Surgery, University Hospital Medical Center “Bežanijska Kosa”, 11070 Belgrade, Serbia; nadj.igor@bkosa.edu.rs (I.N.); milosavljevic.vladimir@bkosa.edu.rs (V.M.); 5 Department of Pathological Anatomy, University Hospital Medical Center “Bežanijska Kosa”, 11070 Belgrade, Serbia; opric.svetlana@bkosa.edu.rs; 6 Department of Healthcare-Associated Infections Prevention, University Hospital Medical Center “Bežanijska Kosa”, 11070 Belgrade, Serbia; 7 Institute of Epidemiology, Faculty of Medicine, University of Belgrade, 11000 Belgrade, Serbia; vladimir.nikolic@med.bg.ac.rs

**Keywords:** breast cancer, sentinel lymph node biopsy, MSKCC nomogram, external validation, predictors, Serbia

## Abstract

**Background/Objectives**: Predicting sentinel lymph node (SLN) involvement in breast cancer can reduce unnecessary axillary lymph node dissections (ALND) and facilitate clinical decision-making. This study aimed to: (i) identify independent predictors for SLN metastasis, and (ii) assess the predictive performance of the MSKCC nomogram for SLN positivity in early-stage breast cancer patients. **Methods**: A retrospective cohort study spanning a six-year period was conducted at a tertiary care oncology department in Belgrade, Serbia. The study included 684 consecutive patients with histologically confirmed primary breast carcinoma, without distant metastasis, who underwent surgical treatment with sentinel lymph node biopsy (SLNB). **Results**: Out of 684 patients, 198 (29%) were SLN-positive. Among the positive cases, 178 (89.9%) had one positive SLN, 19 (9.6%) had two, and 1 (0.5%) had three. Multivariate logistic regression revealed that lymphovascular invasion and tumor sizes >20 mm significantly increased the risk for SLN involvement. Patients with SLN metastasis had significantly higher MSKCC-predicted probabilities compared to SLN-negative patients (52.1 ± 18.5% vs. 33.1 ± 16.9%, *p* < 0.001). The area under the receiver operating characteristic curve (AUC) for the nomogram was 0.778 (95% CI: 0.738–0.818; *p* < 0.001). The nomogram demonstrated acceptable discrimination, with an AUC of 0.778 (95% CI: 0.738–0.818). However, calibration-in-the-large was −0.526 (95% CI: −0.709 to −0.342), indicating systematic overestimation of absolute risk. The calibration slope was 1.152 (95% CI: 0.921–1.383), and the Brier score was 0.174. **Conclusions**: Lymphovascular invasion and primary tumor size >20 mm are the only independent predictors of SLN metastasis. Furthermore, The MSKCC nomogram demonstrated acceptable discrimination but systematically overestimated absolute SLN-metastasis risk. Recalibration and further validation are required before clinical application in this population.

## 1. Introduction

Breast cancer is the most frequent malignant tumor in women worldwide, affecting approximately 1 out of every 20 women in their lifetime. Projections estimate that there will be 3.2 million new breast cancer cases and 1.1 million breast cancer-related deaths annually by 2050 [1]. In Serbia, the public health burden is particularly pronounced, with the average age-adjusted mortality rate for breast cancer increasing from 13.7 per 100,000 in 1985 [2] to 20.6 in 2021 [3]. While breast cancer mortality can be reduced by prevention, early detection, and prompt treatment [4], tailored therapeutic strategies are essential to optimize individual patient outcomes.

One of the main factors influencing the outcome of breast cancer patients is whether the axillary lymph nodes (ALNs) are involved in the malignant process. Axillary lymph node dissection (ALND) was the historical gold standard for staging and treating breast cancer until the late 1990s. Subsequently, the sentinel lymph node biopsy (SLNB) has been widely adopted as the most effective method for evaluating ALN status in patients with clinically node-negative breast cancer [5]. This technique permits the evaluation and removal of only the affected nodes, thereby drastically reducing short- and long-term complications like lymphedema, nerve injury, and shoulder dysfunction, which negatively affect the quality of life [6]. In a recent systematic literature review and meta-analysis, it was estimated that lymphedema was 13.7% more frequent after ALND than after SLNB. Furthermore, the prevalence of postoperative pain was 40% after ALND compared to only 21.7% after SLNB [7].

Axillary management in early breast cancer has progressively shifted toward less invasive approaches. The ACOSOG Z0011 trial demonstrated that completion axillary lymph node dissection (cALND) could be omitted in selected patients with clinically node-negative breast cancer and one or two positive sentinel lymph nodes who underwent breast-conserving surgery and appropriate adjuvant treatment [8]. However, Z0011 did not evaluate omission of sentinel lymph node biopsy (SLNB), because all enrolled patients underwent sentinel node staging.

More recently, the SOUND and INSEMA randomized trials evaluated whether axillary surgery could be omitted altogether in selected patients with clinically node-negative early breast cancer. The SOUND trial demonstrated non-inferiority of no axillary surgery compared with SLNB in patients with breast tumors up to 2 cm and negative preoperative axillary ultrasonography [9]. Similarly, the INSEMA trial supported omission of surgical axillary staging in selected patients with clinically node-negative invasive breast cancer undergoing breast-conserving therapy [10]. These findings have further reduced the need for routine pathological axillary staging in carefully selected patients.

Nevertheless, the results of SOUND and INSEMA do not apply to every patient with early breast cancer. Pathological nodal status may remain relevant in patients who do not meet trial or guideline eligibility criteria, when nodal information could influence locoregional or systemic treatment planning, and when clinicians and patients require an individualized estimate of nodal involvement. Prediction models may therefore complement, but should not replace, established clinical eligibility criteria, axillary imaging, multidisciplinary assessment, and evidence from randomized trials.

The Memorial Sloan Kettering Cancer Center (MSKCC) nomogram was developed to estimate the individual probability of initial SLN metastasis using routinely available clinicopathological variables, including age, tumor size, histological type and grade, tumor location, lymphovascular invasion, multifocality, and hormone receptor status [11]. In the contemporary context of axillary de-escalation, the potential value of such a model lies primarily in individualized risk estimation and counselling, particularly for patients who fall outside the populations evaluated in omission trials. However, before it can be considered for clinical use in a new population, its discrimination, calibration, and clinical utility require external validation. While the MSKCC nomogram has been extensively validated in Western European and North American cohorts, data regarding its performance in Eastern European populations, particularly in Serbia, remain scarce. Furthermore, significant differences in tumor presentation may exist due to suboptimal breast cancer screening coverage. Although the National Breast Cancer Screening Program was adopted by the Government of Serbia in 2009 according to World Health Organization recommendations and officially initiated in 2012/2013, population coverage remains low. Indeed, a recent study demonstrated that only 39.5% of women reported having had a mammogram at least once in their lifetime [12]. This lower screening uptake directly impacts tumor size distribution and stage at diagnosis compared to the Western cohorts in which the MSKCC model was originally developed. Therefore, external validation in specific regional cohorts is critical to confirm the predictive reliability of the MSKCC nomogram before integrating it into local clinical practice. Therefore, the aim of this study was two-fold: (1) to identify independent clinicopathological predictors of SLN metastasis in our cohort; (2) to assess the external validity and predictive performance of the MSKCC nomogram in a Serbian early-stage breast cancer population.

## 2. Materials and Methods

This retrospective cohort study included consecutive patients who underwent breast cancer surgery with sentinel lymph node biopsy at the Department of Surgical Oncology, Clinical Hospital Center Bežanijska kosa, Belgrade, Serbia, between 11 March 2015 and 23 June 2020. Eligible patients had histologically confirmed primary invasive breast carcinoma without distant metastases and were clinically node-negative on physical examination and preoperative axillary imaging, including ultrasonography. Patients who received neoadjuvant systemic therapy before surgery were excluded. Additional exclusion criteria were previous breast cancer surgery, recurrent breast cancer, unavailable SLN pathology results, and insufficient clinicopathological data for meaningful inclusion in the study.

### 2.1. Data Collection and Nomogram Variables

Clinical and pathological data were obtained from institutional medical records. Collected variables included patient age at diagnosis, tumor laterality (right vs. left), tumor localization (classified by breast quadrants and central/axillary positions), tumor size (categorized as <10 mm, 10–20 mm, and >20 mm), histological grade (G1–G3), and histological type. Hormone receptor status (estrogen receptor (ER) and progesterone receptor (PR)), human epidermal growth factor receptor 2 (HER2) expression, Ki67 proliferative index, and intrinsic molecular subtype (Luminal A, Luminal B, HER2-positive, triple-negative) were also assessed. In addition, the type of surgical procedure performed was recorded.

To generate the individual predicted probabilities of initial nodal disease using the online MSKCC Breast Cancer Nomogram tool [11], data were meticulously evaluated across the specific clinicopathological variables required by the model: patient age at diagnosis, pathological tumor size (measured as a continuous variable in cm), histological type (e.g., ductal, lobular), tumor grade (G1–G3), lymphovascular invasion (LVI; present vs. absent), tumor multifocality (present vs. absent), estrogen receptor (ER) status (positive vs. negative), and progesterone receptor (PR) status (positive vs. negative). The primary end point of the predictive validation was the pathologically confirmed presence or absence of macro- or micrometastases within the mapped sentinel lymph nodes.

### 2.2. Histopathological and Immunohistochemical Evaluation

Histological grade was determined according to the Nottingham grading system [13]. Tumor typing followed the World Health Organization [14,15] classification of breast tumors. ER and PR status were determined using immunohistochemistry (IHC), with ≥1% nuclear staining considered positive. HER2 status was evaluated by IHC and categorized on a scale from 0 to 3+. Ki67 expression was reported as a percentage of positively stained tumor cell nuclei, with a cut-off of 20% applied for categorical analyses [13].

### 2.3. Sentinel Lymph Node Biopsy Procedure

SLNB was performed using standard techniques, which included peritumoral or periareolar injection of radiocolloid and/or blue dye for lymphatic mapping. Sentinel nodes were identified intraoperatively and subsequently examined by experienced pathologists. SLN positivity was defined as the presence of macro- or micrometastases.

Patients with positive SLN findings may undergo completion axillary lymph node dissection at the treating surgeon’s discretion.

This study was conducted in accordance with the ethical principles outlined in the Declaration of Helsinki and approved by the Ethics Committee in the hospital (protocol code 12, date of approval 10 January 2019).

### 2.4. Statistical Analysis

Descriptive statistics were used to summarize patient and tumor characteristics. Categorical variables were presented as frequencies and percentages. Continuous variables were presented as means with standard deviations when approximately normally distributed and as medians with ranges otherwise. The distribution of continuous variables was assessed using the Kolmogorov–Smirnov and Shapiro–Wilk tests, together with graphical inspection. Comparisons between patients with negative and positive sentinel lymph node (SLN) findings were performed using the Chi-squared test or Fisher’s exact test for categorical variables and the independent-samples *t*-test or Mann–Whitney U test for continuous variables, as appropriate.

Patients with substantial missing clinicopathological information that prevented meaningful inclusion in the study were excluded. Patients with isolated missing values were retained in the overall descriptive cohort. Missing values were not imputed, and the number of missing observations was reported separately for each variable. Descriptive and univariable analyses were based on the available observations for each variable. Multivariable analyses were conducted as complete-case analyses for the variables included in the respective model.

Univariable logistic regression was used to identify factors associated with SLN positivity. Variables with *p* < 0.10 in the univariable analysis were entered into the initial multivariable logistic regression model. Backward stepwise selection using the Wald method was subsequently applied to obtain the final model, with variables not meeting the retention criterion sequentially removed. Odds ratios (ORs) with 95% confidence intervals (CIs) were reported. The internal validity and stability of the multivariable logistic regression model were assessed using 1000 bootstrap samples. In each bootstrap sample, the complete modeling procedure, including backward Wald variable selection, was repeated. Model performance was evaluated in the bootstrap sample and then in the original dataset. Optimism was estimated as the mean difference between performance in the bootstrap samples and performance obtained when the corresponding bootstrap models were applied to the original dataset. Optimism-corrected estimates were calculated for the AUC, calibration intercept, calibration slope, and Brier score. Predictor-selection frequencies across bootstrap samples were also calculated to assess the stability of the selected predictors. Owing to the small sample size in the Grade 1 subgroup (*n* = 16, 2.3), tumor histological grade was re-categorized into low/intermediate grade (Grade 1 and 2 combined) versus high grade (Grade 3) to avoid unstable parameter estimates during statistical modeling.

For each eligible patient, the probability of SLN metastasis was calculated using the online MSKCC Breast Cancer Nomogram (https://nomograms.mskcc.org/breast/, accessed on 27 January 2025). The model variables included age at diagnosis in years, pathological tumor size in centimeters, histological type, histological grade, tumor location, lymphovascular invasion, multifocality, estrogen receptor status, and progesterone receptor status. Age and tumor size were entered as continuous variables using their original recorded values. Lymphovascular invasion and multifocality were coded as present or absent, while hormone receptor status was coded as positive or negative according to the predefined pathological criteria. Nomogram probabilities were calculated only for patients with complete information for all variables required by the nomogram; missing values were not imputed. The outcome was the histopathologically confirmed presence of macro- or micrometastasis in at least one sentinel lymph node.

Discrimination was evaluated using receiver operating characteristic curve analysis and expressed as the area under the curve (AUC) with a 95% CI. The Youden index was used to identify the threshold that maximized the combined sensitivity and specificity. Sensitivity, specificity, positive predictive value, negative predictive value, and overall classification accuracy were calculated at this threshold. Because the Youden index gives equal weight to false-positive and false-negative classifications and the threshold was derived and evaluated in the same cohort, the resulting cut-off was considered exploratory rather than a clinically validated criterion for omitting SLNB.

Calibration was evaluated using calibration-in-the-large, the calibration slope, and graphical assessment. Calibration-in-the-large was estimated using a logistic regression model with the logit-transformed predicted probability included as an offset; an ideal value is 0. The calibration slope was estimated by regressing observed SLN status on the logit-transformed predicted probability; an ideal value is 1. The calibration plot presented the MSKCC-predicted probability on the *x*-axis and the observed probability of SLN metastasis on the *y*-axis. The plot included a 45° reference line, a loess-smoothed calibration curve with a 95% confidence interval, and observed versus predicted probabilities by risk decile. Overall prediction error was assessed using the Brier score, for which lower values indicate better predictive performance.

Decision-curve analysis was performed in R (version 4.3.2) to evaluate the clinical utility of the nomogram across threshold probabilities from 1% to 50%. At each threshold, patients with a predicted probability equal to or greater than the threshold were classified as candidates for SLNB. Net benefit was calculated using the proportions of true-positive and false-positive classifications, weighted according to the threshold probability, and was compared with biopsy-all and biopsy-none strategies. The net reduction in unnecessary interventions per 100 patients was calculated relative to the biopsy-all strategy. Performance was examined specifically at thresholds of 10%, 23%, and the exploratory Youden-derived threshold of 41.5%. To assess clinical utility, patients were stratified into three predefined risk categories based on their MSKCC-predicted probability of SLN metastasis: low-risk (<30%), intermediate-risk (30–50%), and high-risk (>50%). These cut-off thresholds were chosen a priori based on the literature-established risk stratification protocols in breast cancer management.

All tests were two-sided, and *p* < 0.05 was considered statistically significant. Statistical analyses were performed using SPSS version 26.0 (IBM Corp., Armonk, NY, USA) and R version 4.3.2 (R Foundation for Statistical Computing, Vienna, Austria; https://www.R-project.org/, accessed on 27 January 2025).

## 3. Results

Of the 807 patients who underwent breast cancer surgery and SLNB during the study period, 13 were excluded due to unavailable SLN pathology results; 66 patients were excluded because they had distant metastases, and 44 had incomplete data. Therefore, a total of 684 patients were included in the analysis, of whom 198 (29.0%) had sentinel lymph node positive tests, while 486 (71.0%) had negative SLN findings.

Patient median age was 61 years (range 28–86 years). The main characteristics of the study population are shown in Table 1. A little more than half of the women had luminal A cancer subtype, size 10–20 mm, localized in the right breast, and with a Ki67 index lower than 20%.

Out of all included patients, 198 (29%) had SLN positive, and among them, 178 (89.9%) had one positive SLN while 19 (9.6%) had two and 1 (0.5%) had three positive SLN.

The results of the univariable and multivariable logistic regression analyses are presented in Table 2. In the univariable analysis, age older than 60 years was associated with lower odds of SLN positivity than age 60 years or younger (OR = 0.70, 95% CI: 0.50–0.97; *p* = 0.033). Compared with tumors located in the upper outer quadrant, tumors in the lower outer quadrant (OR = 0.37, 95% CI: 0.16–0.85; *p* = 0.019), lower inner quadrant (OR = 0.37, 95% CI: 0.15–0.95; *p* = 0.038), and upper inner quadrant (OR = 0.33, 95% CI: 0.14–0.78; *p* = 0.011) were associated with lower odds of SLN positivity.

Tumor size was strongly associated with SLN positivity. Compared with tumors smaller than 10 mm, tumors measuring 10–20 mm were associated with higher odds of SLN positivity (OR = 2.07, 95% CI: 1.33–5.10; *p* = 0.005), while tumors larger than 20 mm had an even stronger association (OR = 4.49, 95% CI: 2.25–8.94; *p* < 0.001). In the univariate analysis, tumor histological grade was not a significant predictor of the outcome; when combining Grade 1 and Grade 2 as the reference group, Grade 3 tumors did not show a statistically significant association (OR = 0.991, 95% CI: 0.579–1.696, *p* = 0.974). Consequently, histological grade was not selected for inclusion in the final multivariate model.

The final backward Wald model retained tumor size and LVI. Compared with tumors smaller than 10 mm, tumors measuring 10–20 mm had an adjusted OR of 2.07 (95% CI: 0.98–4.39; *p* = 0.058), while tumors larger than 20 mm had an adjusted OR of 2.76 (95% CI: 1.27–6.02; *p* = 0.011). The presence of LVI was strongly associated with SLN positivity (adjusted OR = 13.42, 95% CI: 8.91–20.19; *p* < 0.001). Age and tumor location were not retained in the final model.

Internal validation was successfully completed in all 1000 bootstrap samples. The apparent AUC of the multivariable model was 0.800, with estimated optimism of 0.013, resulting in an optimism-corrected AUC of 0.787. The optimism-corrected calibration intercept was −0.002, and the optimism-corrected calibration slope was 0.962. The apparent Brier score was 0.143 and the optimism-corrected Brier score was 0.147. LVI was retained in 100% of bootstrap samples and tumor size in 81.1%, whereas age and tumor location were retained in 46.0% and 30.7%, respectively.

Complete data required for MSKCC nomogram validation were available for 659 of the 684 patients. Among these patients, 189 (28.7%) had SLN metastasis and 470 (71.3%) had negative SLN findings. Patients with SLN metastasis had higher MSKCC-predicted probabilities than patients without metastasis (52.1 ± 18.5% vs. 33.1 ± 16.9%, *p* < 0.001; Table 3). The mean predicted probability in the validation cohort was 38.5%, compared with an observed SLN-positivity rate of 28.7%.

**Figure 1:**
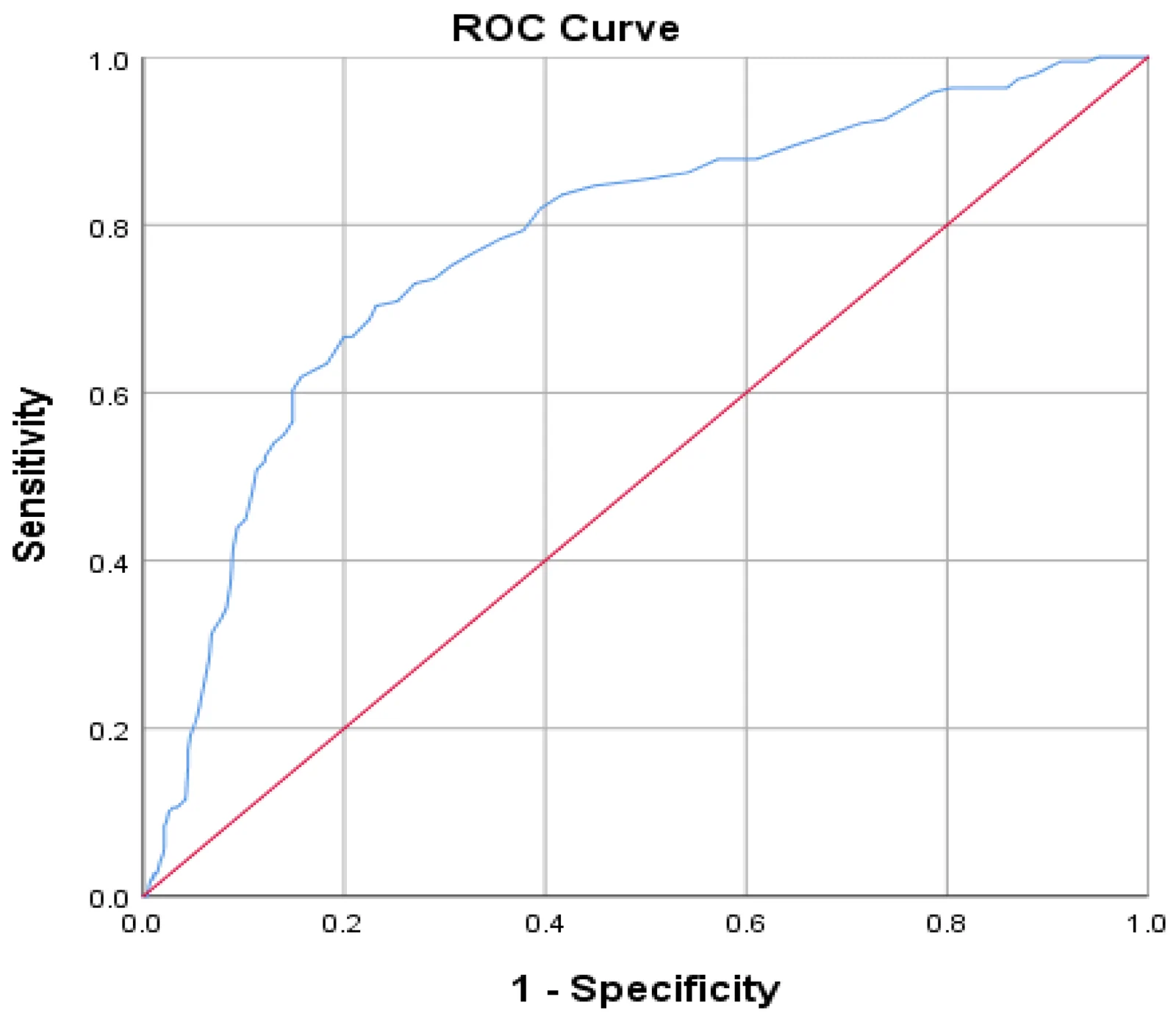
Receiver operating characteristic (ROC) curve of the MSKCC nomogram for predicting sentinel lymph node metastasis. Receiver operating characteristic (ROC) curve of the MSKCC nomogram for predicting sentinel lymph node metastasis. The blue line represents the discriminatory performance of the MSKCC nomogram, while the red diagonal line represents the reference line of no discrimination (AUC = 0.50).

Receiver operating characteristic (ROC) analysis showed that the predicted probability of lymph node spread had good discriminatory ability. The area under the curve (AUC) was 0.778 (95% CI: 0.738–0.818; *p* < 0.001), indicating a statistically significant ability of the model to distinguish between patients with and without sentinel lymph node metastasis (Figure 1).

**Figure 2:**
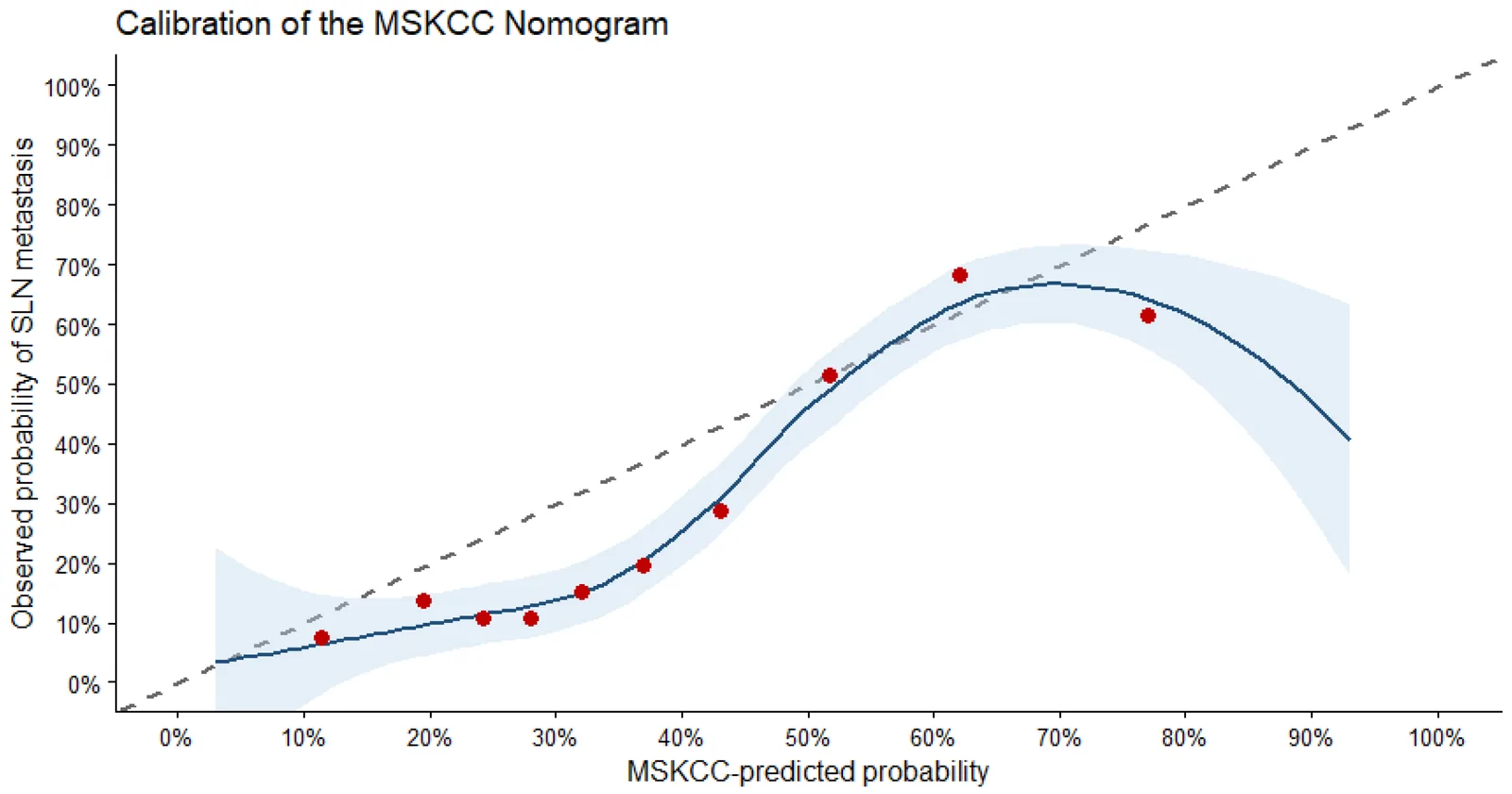
Calibration plot of the nomogram for predicting sentinel lymph node metastasis. The dashed diagonal line represents perfect (ideal) calibration, where the predicted probability exactly matches the observed probability. The solid blue line represents the actual calibration of the MSKCC nomogram in our study population, showing the relationship between predicted and observed probabilities of SLN metastasis.

Calibration-in-the-large was −0.526 (95% CI: −0.709 to −0.342), indicating systematic overestimation of absolute SLN-metastasis risk. The calibration slope was 1.152 (95% CI: 0.921–1.383), with the confidence interval including the ideal value of 1. The Brier score was 0.174. The calibration curve showed that the observed risk was generally lower than the predicted risk across much of the lower and intermediate risk range, whereas estimates at the highest predicted probabilities were less precise (Table 4 and Figure 2).

The Youden-derived threshold was 41.5%, with a sensitivity of 70.4%, specificity of 76.8%, positive predictive value of 55.0%, and negative predictive value of 86.6%. At this threshold, 133 of 189 patients with SLN metastasis were correctly classified, whereas 56 (29.6%) were classified below the threshold. Because this threshold was derived and evaluated in the same cohort and does not account for the unequal clinical consequences of false-positive and false-negative classifications, it was considered exploratory.

**Figure 3:**
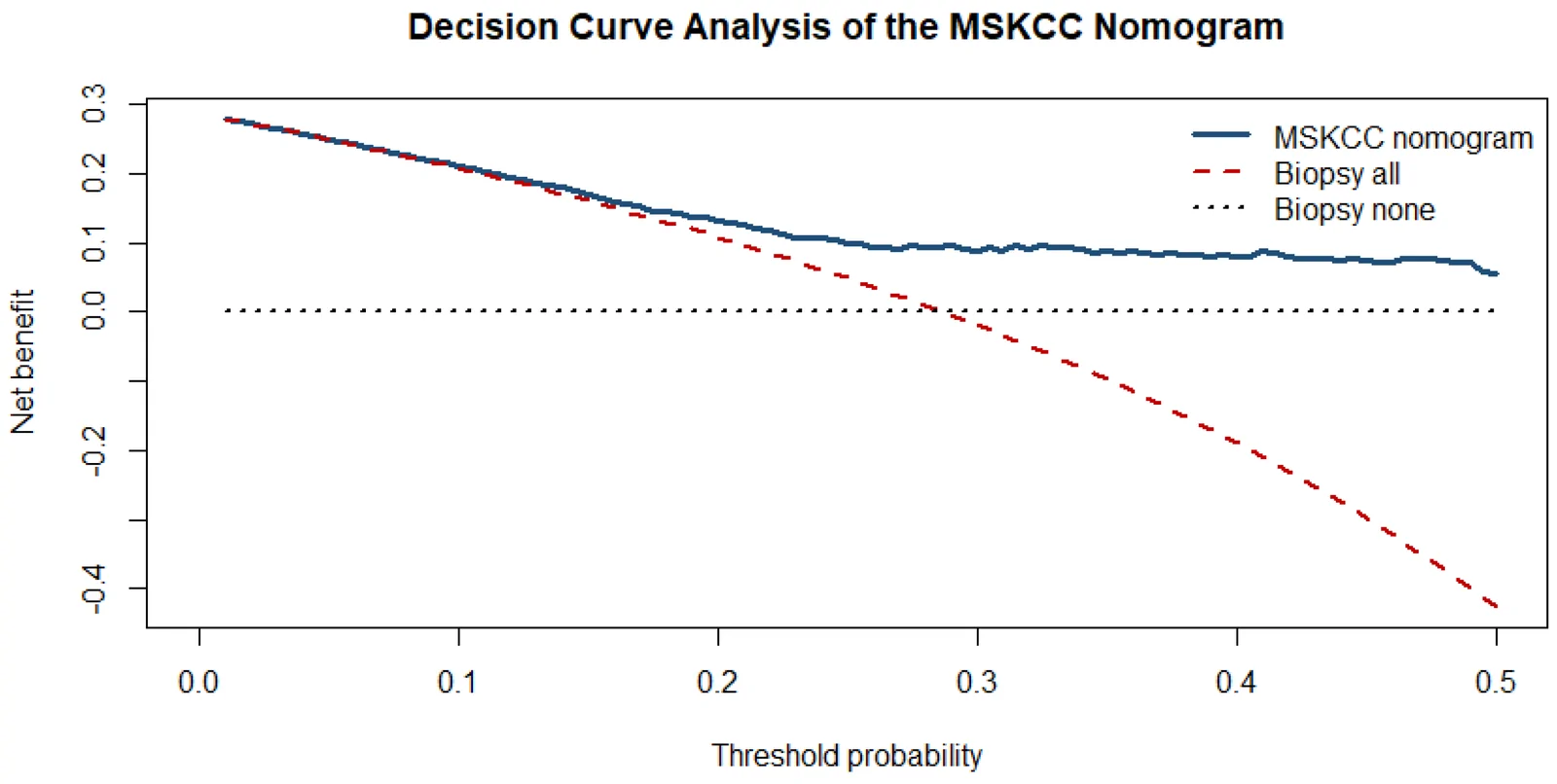
Decision-curve analysis of the MSKCC nomogram for predicting sentinel lymph node metastasis.

Decision-curve analysis showed that the nomogram provided a greater net benefit than both biopsy-all and biopsy-none strategies across threshold probabilities from 3.5% to 50% (Figure 3). At a threshold of 10%, sensitivity was 100.0% and specificity was 4.9%, corresponding to an estimated net reduction of 3.5 unnecessary biopsies per 100 patients compared with the biopsy-all strategy. At a threshold of 23%, sensitivity was 92.6% and specificity was 26.2%, with an estimated net reduction of 11.6 biopsies per 100 patients. At the exploratory threshold of 41.5%, sensitivity was 70.4% and specificity was 76.8%, with an estimated net reduction of 42.8 biopsies per 100 patients. However, 56 of 189 patients with SLN metastasis were classified below the 41.5% threshold.

Patients were stratified into three predefined, literature-based risk categories according to their predicted probability of sentinel lymph node metastasis. The rate of sentinel lymph node metastasis increased markedly across risk categories: approximately 10.6% in the low-risk group, 24.8% in the intermediate-risk group, and 61.2% in the high-risk group (Table 5). This gradient of risk was statistically significant (χ^2^ = 130.3, *p* < 0.001).

## 4. Discussion

The results of our study demonstrate that lymphovascular invasion (LVI) and primary tumor size >20 mm are the only independent predictors of sentinel lymph node (SLN) metastasis in the final multivariate logistic regression model. Notably, LVI emerged as the single most powerful predictor of nodal positivity, exhibiting a massive effect size in both univariate (OR = 14.43, *p* < 0.001) and multivariate (OR = 13.41, *p* < 0.001) environments. Conversely, variables such as age and specific tumor quadrants, which initially demonstrated statistical significance in the univariate analysis, lost their independent predictive power after adjusting for LVI and tumor size. Our finding that LVI is heavily associated with axillary nodal involvement aligns with a vast body of oncology literature and recent meta-analyses [16]. Only a significantly higher proportion of patients with SLN metastasis had LVI, which is in concordance with other studies [17,18]. Based on the results of the meta-analysis, it can be suggested that preoperative evaluation of LVI with a non-invasive and convenient tool, such as MRI, can predict LVI and make a choice for better therapy choices [19,20]. The presence of tumor cells within endothelial-lined lymphatic or vascular channels represents a crucial biological milestone in structural dissemination, directly facilitating the transit of breast cancer cells to the regional nodal basin.

Tumor size is a powerful predictor of SLN metastasis and directly impacts the SLN metastasis rate [21]. In our cohort, while tumors between 10 and 20 mm demonstrated a trend toward higher risk, only primary tumors larger than 20 mm retained independent statistical significance in the multivariable model (OR = 2.76, *p* = 0.011). This observation is consistent with historical surveillance data, including data from the recent population-level analyses evaluating data from the Surveillance, Epidemiology, and End Results (SEER) registry that identified primary tumor size as one of the nine most reliable predictive variables for SLN metastasis [22]. Larger tumor volumes inherently harbor a higher probability of expansive invasive components and occult LVI, compounding the overall risk of metastatic spread. Tumors smaller than 10 mm have a relatively low risk of SLN metastasis, less than 10%, while tumors larger than 20 mm increase the risk [17]. Larger tumors have a higher likelihood of harboring invasive components and lymphatic vessel invasion (LVI), which directly increases the probability of cancer cells spreading to the lymph nodes [23,24]. It was revealed that besides tumor size and LVI, tumor location and biological subtypes, like triple-negative aggressive form, have a significant effect on metastasis risk, local recurrence, and patient survival [23,25].

In contrast, age (>60 years) and specific anatomical quadrants (lower outer, lower inner, and upper inner) were significantly associated with lower SLN positivity only in the univariate analysis. The univariate model showed that patients older than 60 years had a lower risk of regional metastasis (OR = 0.70, *p* = 0.033). Several studies have found that positive SLN metastasis is significantly lower in older breast cancer patients compared to younger ones [17,26,27,28,29], although various age cut-offs (ranging from 40 to 70 years) have been utilized across institutions [26,27,28,29] but with different cut-points of age. In some studies, it was 40 years [27], 50 years [26], 60 years [29], or 70 years [28]. In a retrospective population-based study that included about 25 hundred patients, younger patients (<40 years) frequently present with distinct biological phenotypes, including lower estrogen receptor (ER) and progesterone receptor (PR) expression, more advanced TNM stages at diagnosis, and a worse overall prognosis [30]. Because tumors in older women frequently display less aggressive biological features and lower proliferation rates, consensus guidelines and international clinical campaigns—such as the US Choosing Wisely initiative and the American Society of Clinical Oncology (ASCO) updates—recommend de-escalating or entirely omitting routine SLN biopsy in specific low-risk elderly populations [31,32,33].

Bootstrap internal validation indicated only modest optimism in the multivariable model, with the AUC decreasing from 0.800 to 0.787 after optimism correction. The optimism-corrected calibration slope of 0.962 and calibration intercept of −0.002 were close to their ideal values of 1 and 0, respectively. LVI was retained in every bootstrap sample, supporting its stability as the strongest predictor, while tumor size was retained in 81.1% of samples. In contrast, age and tumor location demonstrated lower selection frequencies, indicating that their associations were less stable after accounting for resampling variability. Nevertheless, these findings represent internal validation within a single-center cohort, and external validation would be required before the regression model could be used for individual risk prediction.

Similarly, specific tumor locations and quadrant-based distributions of multifocality or multicentricity showed significant protective trends in the univariate analysis com-pared to the upper outer quadrant, but failed to sustain independent significance in the multivariable environment. A single term for multifocal and multicentric breast cancer is often used, although there are slight differences in meaning. However, quadrant-based definitions of multifocality/multicentricity currently lack prognostic or therapeutic significance and serve only to aid surgical planning [34]. Furthermore, other aggressive biological characteristics in our study, such as triple-negative subtypes or high Ki67 (>20%), showed no significant distribution differences between the SLN-positive and SLN-negative groups, underlining the overwhelming dominant effect of structural parameters like LVI and size in this cohort.

The MSKCC nomogram demonstrated acceptable discrimination in our cohort, with an AUC of 0.778. An AUC between 0.70 and 0.80 is generally interpreted as indicating acceptable discriminatory performance [35,36]. The MSKCC nomogram was originally developed by Bevilacqua et al., with an AUC of 0.75 [11]. Subsequent external validation studies reported variable performance, including an AUC of 0.67 in a Dutch population and values of approximately 0.77–0.80 in other cohorts [37,38,39]. Our findings therefore support the ability of the nomogram to distinguish between patients with higher and lower probabilities of SLN metastasis. However, acceptable discrimination does not necessarily imply accurate estimation of absolute risk, as discrimination and calibration rep-resent different aspects of predictive performance [40].

In our validation cohort, the mean MSKCC-predicted probability was 38.5%, whereas the observed SLN-positivity rate was 28.7%. Calibration-in-the-large was −0.526 (95% CI: −0.709 to −0.342), confirming systematic overestimation of absolute risk. The calibration slope was 1.152 (95% CI: 0.921–1.383), with the confidence interval including the ideal value of 1. Therefore, the principal calibration problem appeared to be a general upward shift in predicted probabilities rather than clear evidence of an inappropriate spread of predictions. The calibration curve similarly demonstrated overprediction across much of the lower and intermediate risk range, while estimates at the highest predicted probabilities were less precise. Consequently, the original MSKCC probabilities should not be directly applied to clinical decision-making in this population without recalibration and subsequent validation [40].

Previous studies have evaluated conservative thresholds, including probabilities of approximately 10% and 23%, to identify patients at lower risk of SLN metastasis [41]. In the present study, the Youden index identified 41.5% as the threshold that maximized the combined sensitivity and specificity. However, the Youden index assigns equal im-portance to false-positive and false-negative classifications and does not account for their different clinical consequences. At the 41.5% threshold, sensitivity was 70.4%, meaning that 56 of 189 patients with SLN metastasis were classified below the threshold. Further-more, the negative predictive value of 86.6% indicates that approximately one in seven patients classified below the threshold nevertheless had SLN metastasis. Accordingly, the 41.5% threshold should be considered an exploratory statistical cut-off and not a clinically validated criterion for omitting SLNB.

Decision-curve analysis provides additional information about clinical utility by incorporating the relative consequences of false-positive and false-negative classifications across different threshold probabilities [42]. In our study, the nomogram provided greater net benefit than biopsy-all and biopsy-none strategies across threshold probabilities from 3.5% to 50%. Nevertheless, the clinical implications differed substantially according to the selected threshold. At a threshold of 10%, sensitivity was 100.0%, but specificity was only 4.9%, and the estimated net reduction was 3.5 biopsies per 100 patients compared with the biopsy-all strategy. At 23%, sensitivity was 92.6%, specificity was 26.2%, and the estimated net reduction was 11.6 biopsies per 100 patients. At 41.5%, the estimated net reduction increased to 42.8 biopsies per 100 patients, but 29.6% of patients with SLN metastasis were classified below the threshold. These findings demonstrate the trade-off between reducing interventions and failing to identify nodal metastases. A positive net benefit at a particular threshold does not, by itself, establish that omission of SLNB is oncologically safe.

All patients included in the present cohort underwent SLNB. Therefore, patients classified below a particular probability threshold still had their sentinel lymph nodes examined and received clinical management based on the pathological findings. In addition, longitudinal follow-up of patients managed without SLNB was not available. Consequently, this study cannot evaluate axillary recurrence or other oncological outcomes following omission of SLNB. Evidence supporting the omission of axillary surgery in select-ed patients derives from prospective randomized trials, including SOUND and INSEMA, rather than from a nomogram threshold alone [9,10].

It is also important to distinguish omission of completion ALND from omission of SLNB. Decisions regarding completion ALND are made after nodal status has been established, whereas omission of SLNB leaves pathological nodal status unknown. Our findings evaluate the probability of SLN positivity and do not provide evidence supporting the omission of either procedure. Similarly, the current ASCO recommendation applies to carefully selected patients meeting defined clinical criteria, including age, tumor size, histological grade, hormone receptor and HER2 status, negative preoperative axillary ultra-sonography, and planned breast-conserving treatment [33]. This clinical eligibility rule should not be interpreted as validation of the 41.5% nomogram threshold.

Taken together, our findings indicate that the MSKCC nomogram provides acceptable discrimination but systematically overestimates absolute SLN-metastasis risk in this Serbian population. Adjustment of the model intercept may improve calibration because the calibration slope was reasonably close to its ideal value. However, any recalibrated model would require additional internal validation and preferably prospective external validation before being used to guide decisions regarding axillary surgery [40].

The contemporary role of predicting SLN status must be considered in the context of evidence supporting omission of axillary surgery in selected patients. For patients who satisfy SOUND, INSEMA, or guideline-based eligibility criteria, a nomogram may provide limited additional value if pathological nodal status is not expected to alter treatment. Its potential relevance may be greater among patients who fall outside these eligibility criteria or when the estimated probability of nodal involvement could inform multidisciplinary discussion, patient counselling, radiotherapy planning, or systemic treatment decisions. Nevertheless, a prediction model should not be used as a substitute for trial-based eligibility criteria or as a standalone justification for omitting SLNB. In our cohort, systematic overestimation of absolute risk further limits direct clinical application of the original MSKCC probabilities without recalibration and prospective validation.

This study has several limitations that should be considered when interpreting our findings. First, its retrospective single-center design introduces inherent risks of selection bias, particularly regarding patients excluded due to missing clinical or pathological variables, and limits the generalizability of our findings to other regions. Second, conducting the study over a six-year period carries the potential for temporal bias and subtle practice drift in SLNB techniques, pathological processing, or surgeon experience; however, temporal validation across distinct time frames was not performed. Third, while the MSKCC nomogram demonstrated satisfactory discrimination in our cohort, we did not perform formal model recalibration (adjusting the intercept or calibration slope) specifically for the baseline risk of the Serbian population, which may be necessary before direct integration into local clinical practice. Further, retrospective study design has well-known limitations such as missing clinical data that were originally not designed to collect data for research and bias in target demographics. Finally, long-term survival and recurrence outcomes were not assessed, as the study focused primarily on perioperative staging accuracy and clinicopathological predictors of SLN involvement. Future prospective, multicenter studies with formal recalibration and longitudinal follow-up are warranted to address these limitations.

## 5. Conclusions

This study identifies lymphovascular invasion (LVI) and a primary tumor size greater than 20 mm as the only independent predictors of SLN metastasis in breast cancer, with LVI emerging as the most powerful and stable biological driver of nodal positivity.

Additionally, the MSKCC nomogram demonstrated good accuracy in predicting overall sentinel lymph node metastasis in the Serbian breast cancer population. These findings can help clinicians to preoperatively estimate the extent of axillary tumor, which supports the trend toward a personalized and less invasive surgical approach.

## Figures and Tables

**Table 1:** Clinicopathological characteristics of the study population.

	Total *n*	%
Total	684	100.0
Age (years)		
≤60	319	46.6
>60	365	53.4
Laterality of tumor		
Right	350	51.2
Left	334	48.8
Tumor location		
Upper outer quadrant	292	42.7
Lower outer quadrant	73	10.7
Upper inner quadrant	160	23.4
Lower inner quadrant	58	8.5
Central (Nipple area)	68	9.9
More than one quadrant	25	3.6
Missing localization	8	1.2
Tumor size (mm)		
<10 mm	88	12.9
10–20 mm	375	54.8
>20 mm	215	31.4
Missing size	6	0.9
Histological grade		
G1	15	2.2
G2	587	85.8
G3	72	10.5
Missing grade	10	1.5
Histological type		
Invasive ductal carcinoma (ICD)	535	78.3
Invasive lobular carcinoma (ILC)	91	13.4
Mucinous carcinoma	24	3.5
Tubular carcinoma	14	2.0
Papillary carcinoma	14	2.0
Paget’s disease	1	0.1
Missing type	5	0.7
Estrogen receptor (ER)		
Negative	70	10.2
Positive	611	89.4
Missing estrogen receptor	3	0.4
Progesterone receptor (PR)		
Negative	118	17.3
Positive	563	82.3
Missing progesterone receptor	3	0.4
HER		
0	588	86.0
1	39	5.7
2+	51	7.4
3+	2	0.3
Missing HER2	4	0.6
Molecular subtype		
Luminal A	364	53.2
Luminal B	227	33.2
HER2-positive	28	4.1
Triple-negative	61	8.9
Missing subtype	4	0.6
Ki67 index—median (min–max)	20% (1–90%)	1–90%
≤20%	381	55.7
>20%	300	43.9
Missing Ki67 index	3	0.4
Lymphovascular invasion		
No	495	72.4
Yes	189	27.6
Multifocality		
No	643	94.0
Yes	41	6.0

**Table 2:** Univariate and multivariate logistic regression analysis of factors associated with sentinel lymph node positivity.

	Univariate Logistic Regression OR (95% CI)	*p* Value	Multivariate Logistic Regression OR (95% CI)	*p* Value
Age (years)				
≤60				
>60	**0.70 (0.50–0.97** **)**	**0.033**		
Laterality of tumor				
Right				
Left	0.99 (0.71–1.37)	0.939		
Location				
Upper outer quadrant	Ref.			
Lower outer quadrant	**0.37 (0.16–0.85)**	**0.019**		
Lower inner quadrant	**0.37 (0.15–0.95)**	**0.038**		
Upper inner quadrant	**0.33 (0.14–0.78)**	**0.011**		
Central quadrant	0.38 (0.15–1.01)	0.052		
More than one quadrant	0.41 (0.16–1.05)	0.064		
Tumor size (mm)				
<10 mm	**Ref.**		**Ref.**	
10–20 mm	**2.61 (1.33–5.10)**	**0.005**	**2.07 (0.98–4.39)**	**0.058**
>20 mm	**4.49 (2.25–8.94)**	**<0.001**	**2.76 (1.27–6.02)**	**0.011**
Histological grade				
G1	Ref.			
G2	1.15 (0.36–3.66)	0.814		
G3	1.13 (0.32–3.96)	0.846		
Histological type				
Invasive ductal carcinoma (ICD)	Ref.			
Invasive lobular carcinoma (ILC)	0.70 (0.42–1.16)	0.167		
Mucinous carcinoma	0.44 (0.15–1.30)	0.136		
Tubular carcinoma	0.17 (0.02–1.29)	0.087		
Papillary carcinoma	0.34 (0.07–1.51)	0.154		
Paget’s disease	NA			
Estrogen receptor (ER)				
Negative				
Positive	1.11 (0.64–1.93)	0.713		
Progesterone receptor (PR)				
Negative				
Positive	1.31 (0.83–2.07)	0.242		
HER				
0	Ref.			
1	1.57 (0.80–3.06)	0.189		
2+	0.95 (0.50–1.80)	0.871		
3+	2.51 (0.16–40.30)	0.517		
Molecular subtype				
Luminal A	Ref.			
Luminal B	1.13 (0.78–1.62)	0.522		
HER2-positive	1.22 (0.53–2.79)	0.635		
Triple-negative	1.08 (0.59–1.96)	0.802		
Ki67				
≤20%				
>20%	1.09 (0.78–1.52)	0.622		
Lymphovascular invasion	14.43 (9.65–21.57)	<0.001	**13.41 (8.91–20.19)**	**<0.001**

Statistically significant values are highlighted in bold.

**Table 3:** MSKCC breast cancer nomogram.

	Total (Mean ± SD)	Sentinel Negative (Mean ± SD)	Sentinel Positive (Mean ± SD)	*p* Value
MSKCC Breast Cancer Nomogram: Sentinel Lymph Node Metastasis	38.5 ± 19.4	33.1 ± 16.9	52.1 ± 18.5	<0.001

MSKCC—Memorial Sloan Kettering Cancer Center.

**Table 4:** Calibration of the breast cancer nomogram.

Decile of Predicted Risk	Predicted Probability	Observed Metastasis
1	11.4%	8%
2	19.7%	13%
3	24.6%	13%
4	28.4%	8%
5	32.3%	18%
6	36.8%	19%
7	43.0%	29%
8	51.6%	52%
9	61.9%	66%
10	77.0%	62%

**Table 5:** SLN metastasis rate across risk groups.

Risk Group (% of Probability)	Patients	SLN Metastasis	Metastasis Rate	*p* Value
Low risk (<30%)	255	27	10.6%	<0.001
Intermediate risk (30–50%)	234	58	24.8%	
High risk (>50%)	170	104	61.2%	

## Data Availability

The data presented in this study are available on request from the corresponding author due to (privacy and ethical reasons).

## Abbreviations

The following abbreviations are used in this manuscript:

ACOSOG	American College of Surgeons Oncology Group
ALN	Axillary lymph node
ASCO	American Society of Clinical Oncology
AUC	Area under the curve
cALND	Completion axillary lymph node dissection
CI	Confidence interval
ER	Estrogen receptor
HER2	Human epidermal growth factor receptor 2
IHC	Immunohistochemistry
LVI	Lymphovascular invasion
MRI	Magnetic resonance imaging
MSKCC	Memorial Sloan Kettering Cancer Center
OR	Odds ratio
PR	Progesterone receptor
ROC	Receiver operating characteristic
SEER	Surveillance, Epidemiology, and End Results
SLN	Sentinel lymph node
SLNB	Sentinel lymph node biopsy
SPSS	Statistical Package for the Social Sciences
TNM	Tumor–node–metastasis
